# A mixed-method investigation of patient monitoring and enhanced feedback in routine practice: Barriers and facilitators

**DOI:** 10.1080/10503307.2015.1051163

**Published:** 2015-10-05

**Authors:** Mike Lucock, Jeremy Halstead, Chris Leach, Michael Barkham, Samantha Tucker, Chloe Randal, Joanne Middleton, Wajid Khan, Hannah Catlow, Emma Waters, David Saxon

**Affiliations:** ^a^ South West Yorkshire Partnership NHS Foundation Trust, UK; ^b^ Centre for Applied Psychological and Health Research, University of Huddersfield, Huddersfield,UK; ^c^ Clinical Psychology, University of Leeds, Leeds,UK; ^d^ Department of Psychology, Centre for Psychological Services Research, University of Sheffield, Sheffield,UK; ^e^ School of Psychological Sciences, University of Manchester, Manchester,UK

**Keywords:** psychological therapies, outcomes monitoring, feedback, implementation

## Abstract

**Objective**: To investigate the barriers and facilitators of an effective implementation of an outcome monitoring and feedback system in a UK National Health Service psychological therapy service. **Method**: An outcome monitoring system was introduced in two services. Enhanced feedback was given to therapists after session 4. Qualitative and quantitative methods were used, including questionnaires for therapists and patients. Thematic analysis was carried out on written and verbal feedback from therapists. Analysis of patient outcomes for 202 episodes of therapy was compared with benchmark data of 136 episodes of therapy for which feedback was not given to therapists. **Results**: Themes influencing the feasibility and acceptability of the feedback system were the extent to which therapists integrated the measures and feedback into the therapy, availability of administrative support, information technology, and complexity of the service. There were low levels of therapist actions resulting from the feedback, including discussing the feedback in supervision and with patients. **Conclusions:** The findings support the feasibility and acceptability of setting up a routine system in a complex service, but a number of challenges and barriers have to be overcome and therapist differences are apparent. More research on implementation and effectiveness is needed in diverse clinical settings.

## Abstract (it)

Obiettivo: studiare gli ostacoli e i facilitatori di una efficace attuazione di un monitoraggio degli esiti e di un sistema di feedback in un servizio di terapia psicologica del Servizio Sanitario Nazionale del Regno Unito. Metodo: è stato introdotto un sistema di monitoraggio dell'esito in due servizi. E’ stato fornito un maggior feedback ai terapeuti dopo la seduta 4. E’ stato usato un metodo qualitativo e quantitativo, tra cui questionari per i terapeuti e pazienti. E’ stata effettuata un'analisi tematica sui feedback scritti e verbali dei terapeuti. L'analisi degli esiti dei pazienti a partire da 202 episodi della terapia sono stati confrontati con i risultati standard di 136 episodi di terapia di soggetti per i quali non erano stati dati feedback al terapeuta. Risultati: temi che influenzano la fattibilità e l'accettabilità del sistema di feedback sono stati la misura in cui i terapeuti integravano le misure e feedback nella terapia, la disponibilità di supporto amministrativo, informazioni tecniche e la complessità del servizio. Ci sono stati bassi livelli di azioni del terapeuta derivanti dal feedback, tra cui discutere il feedback in supervisione e con i pazienti. Conclusioni: I risultati confermano la fattibilità e l'accettabilità della costruzione di un sistema di routine in un servizio complesso, ma una serie di sfide e ostacoli devono essere superati e le differenze tra terapeuti sono evidenti. E’ necessaria una maggiore ricerca sull'attuazione e sull'efficacia in diversi contesti clinici.

## Abstract (de)

**Zusammenfassung: Ziele:** Ziel war es, Barrieren und unterstützende Faktoren einer effektiven Implementierung eines Outcome Monitoring- und Feedbacksystems in einem psychotherapeutischen Dienst des britischen nationalen Gesundheitssystems zu untersuchen. **Methode:** Ein Outcome Monitoring System wurde bei zwei Diensten eingeführt. Den Therapeuten wurde erweitertes Feedback nach der 4. Sitzung gegeben. Qualitative und quantitative Methoden wurden genutzt, einschließlich Fragebögen für Therapeuten und Patienten. Die thematischen Analysen wurden anhand von schriftlichen und mündlichen Rückmeldungen der Therapeuten durchgeführt. Analysen der Patientenergebnisse für 202 therapeutische Einheiten wurde mit Benchmarkdaten von 136 Therapieeinheiten verglichen, bei denen dem Therapeuten keine Rückmeldung gegeben wurde. **Ergebnisse:** Themen, welche die Durchführbarkeit und Akzeptanz des Feedbacksystems beeinflussten, waren das Ausmaß in dem Therapeuten die Instrumente und Rückmeldungen in die Therapie integrierten, die Verfügbarkeit der administrativen Unterstützung, die Informationstechnologie und die Komplexität des Dienstes. Durch das Feedback ergaben sich geringe Veränderungen des therapeutischen Verhaltens, welche sich in der Besprechung der Rückmeldungen im Rahmen von Supervision oder mit dem Patienten äußerten. **Schlussfolgerung:** Die Untersuchung spricht für eine Durchführbarkeit und Akzeptanz der Einführung eines Routinesystems in einem komplexen Dienst, jedoch müssen eine Reihe von Herausforderungen und Barrieren überwunden werden und Unterschiede zwischen Therapeuten sind erkennbar. Es bedarf weiterer Untersuchungen zur Implementierung und Effektivität in unterschiedlichen klinischen Settings.

## Abstract (pt)

**Objetivo:** Investigar as barreiras e os facilitadores do resultado da implementação efetiva de um sistema de *feedback* no serviço de terapia psicológica do Serviço Nacional de Saúde do Reino Unido. **Método:** Foi introduzido um sistema de monitorização de resultados em dois serviços. Após a quarta sessão foi dado um reforço de *feedback* aos terapeutas. Foram utilizados métodos qualitativos e quantitativos, incluindo questionários para os terapeutas e clientes. Foi realizada uma análise temática acerca dos dados de *feedback* escritos e verbalizados pelos terapeutas. A análise dos resultados dos clientes para 202 episódios da terapia foi comparada com dados de referência de 136 episódios de terapia sobre os quais não foi dado qualquer *feedback* aos terapeutas. **Resultados:** Os temas que influenciaram a viabilidade do sistema de *feedback* resultaram do modo como os terapeutas integraram as medidas e o *feedback* nas suas ações terapêuticas, desde o *feedback* até à discussão do *feedback* na supervisão e com os pacientes. **Conclusões:** Os resultados apoiam a viabilidade de montar um sistema de rotina num serviço tão complexo, mas é necessário ultrapassar uma série de desafios e barreiras, e as diferenças nos terapeutas são evidentes. É necessária mais investigação acerca da implementação e eficácia em diversos contextos clínicos.

## 摘要

目的：探討在一英國國家健康機構之心理治療服務中，有效運用效果監控與回饋系統的阻礙及促進因子。方法：將效果監控引入二個機構中，同時在第四次晤談後給予治療師增強的回饋。同時運用質化及量化方法，包括給治療師及病人進行問卷評估。對治療師的書面及口語回饋進行主題分析。比較治療師有接受回饋的病人之202段治療事件與治療師沒有接受回饋的病人之136段治療事件的治療效果差異。結果：治療師將評量及回饋納入治療過程的程度、行政支持的可得性、資訊科技及服務的複雜性，是影響回饋系統的可行性與可接受性之重要主題。但僅有極少量的治療師行為是導自於此回饋，包括與督導或病人討論這些回饋。結論：研究結果支持在一個複雜的服務體系中建構一個例行系統的可行性與可接受性，但需要克服很多的挑戰與阻礙因素，也顯見不同治療師的差異。未來需對不同機構進行更多應用及有效性的研究。

關鍵字：心理治療；效果監控；回饋；應用

## Introduction

There is substantial evidence for the efficacy and effectiveness of psychological therapies (Lambert, 2013). Despite this body of literature, a significant number of patients do not benefit. Hansen, Lambert, and Forman (2002) report that about a third of patients receiving psychotherapy in RCTs either show no benefit or deteriorate. The rates of poor response are reported to be higher in routine services, with Hansen et al. (2002) reporting 56% of patients making no reliable change across studies in routine practice involving over 6000 patients with an additional average reliable deterioration rate of 8%. The importance of deterioration in psychotherapy has been highlighted (e.g., Lilienfeld, 2007) and Mohr (1995), in a review of 46 studies, identified patient, therapist, and therapy variables associated with negative outcome. The patient characteristics identified included borderline personality, obsessive–compulsive problems with severe interpersonal difficulties, poor motivation, and those patients expecting psychotherapy to be painless. This evidence underlines the importance of identifying patients at risk of a poor treatment response.

There is now widespread acknowledgment that an important way of improving outcomes involves monitoring the progress of patients during therapy and providing timely feedback on the monitoring data to therapists. This activity has yielded a burgeoning literature comprising texts (e.g., Lambert, 2010), reviews (e.g., Carlier et al., 2012), special issues (e.g., Fitzpatrick, 2012), and opinion pieces (e.g., Macdonald & Mellor-Clark, 2015). Monitoring progress and providing feedback is consistent with the traditions of patient-focused research (Howard, Moras, Brill, Martinovich, & Lutz, 1996) and the scientist practitioner approach (Hayes, Barlow, & Nelson, 1999). However, only in recent years have patient monitoring and feedback systems become embedded in routine practice. Examples are Lambert's work using the Outcome Questionnaire (Lambert et al., 2004); the Clinical Outcomes in Routine Evaluation (CORE) OM and CORE system in the UK (Barkham et al., 2010); and Miller and Duncan's development and implementation of two 4-item measures of treatment progress and therapeutic alliance (Miller, Duncan, Sorrell, & Brown, 2005). Part of the rationale for using these systems rests on evidence that early response to therapy predicts outcome (Lutz et al., 2006; Lutz, Stulz, & Köck, 2009; Stulz, Lutz, Leach, Lucock, & Barkham, 2007). In particular, there is evidence that poor early response to therapy is a predictor of a poor outcome (Kuyken, 2004; Lambert et al., 2002). Another justification for patient monitoring and feedback systems is the evidence that therapists are poor judges of their patients’ outcomes (Garb, 2005; Sapyta, Reimer, & Bickman, 2005). Moreover, they are poor at predicting which of their patients are likely to deteriorate (Hannan et al., 2005). This finding is consistent with long-standing evidence that clinical judgement can be relatively poor and inferior to statistical predictions (Dawes, Faust, & Meehl, 1989; Meehl, 1954). Tracey, Wampold, Lichtenberg, and Goodyear (2014) argue that a further justification for attending to feedback about patients’ progress is that it is a necessary but not sufficient requirement for developing the expertise of therapists.

Lambert and colleagues introduced the notion of patients who are “not on track” (NOT) as a means of identifying patients who are not achieving the expected recovery trajectory and therefore at risk of treatment failure. They found that providing feedback to therapists on the progress of these patients improved outcomes (Lambert et al., 2001, 2002). The addition of a set of clinical support tools, which provided information to therapists about the quality of the therapeutic relationship, the patient's social support, motivation, and experience of life events, further improved outcomes for NOT patients (Whipple et al., 2003). There is further evidence of moderate effects of feedback on outcome from other settings and countries (e.g., Byrne, Hooke, Newnham, & Page, 2012; Hansson, Rundberg, Ö sterling, Ö jehagen, & Berglund, 2013; Probst et al., 2013).

Set against this promising body of evidence, a recent independent review of patient outcome feedback systems by Davidson, Perry, and Bell (2015) heeded caution in relation to the extent to which findings could be generalized to non-US, and specifically UK, settings. The authors note that only the later studies—post-2009—are likely to represent the diversity and severity of patients seen in the UK National Health Service (NHS) and the impact of feedback in these studies appears to be reduced. Accordingly, the present study investigated the feasibility of setting up an outcome feedback system within a routine UK NHS service setting that met the needs of a more diverse and broad spectrum of patient needs.

Despite this growing body of evidence, there have been few studies investigating mediators related to feedback systems, such as therapist behaviour (e.g., how they act on the feedback) or organizational factors. Boswell, Kraus, Miller, and Lambert (2015) discuss a number of these factors and identify some of the benefits, obstacles, and challenges associated with routine outcome monitoring based on their experience with different systems over many years. They identify issues such as time burden, multiple stakeholders with different needs, and turnover of staff, particularly local “champions,” as well as fear and mistrust by therapists. Further research is required to understand the contribution of such factors to feedback, which might then inform improved methods for presenting feedback, engaging therapists, and understanding how clinicians make use of the feedback provided to them (Sapyta et al., 2005). Boswell et al. (2015) point out that despite research on feedback in psychotherapy, more needs to be learned about the implementation and sustained use of outcome monitoring and feedback systems in order to improve adoption and compliance. This should involve identifying organizational issues that may act as barriers to and facilitators of implementing patient monitoring and feedback systems in routine services. Services will vary in a number of key aspects, all of which may impact on the feasibility of running an effective feedback system. This situation is likely to be more important in complex services, for example, with multiple clinical bases, where a range of therapies is provided (including group work) and where patients receive more than one episode of therapy. Hence, the present study aimed to identify the barriers and facilitators to effective implementation and clinician engagement within a complex routine UK service setting.

The current study therefore had three specific aims: (1) to assess the acceptability and feasibility, including organizational factors, of running an outcomes monitoring and feedback system in a routine UK NHS psychological therapy service; (2) to investigate if and how clinicians make use of feedback; and (3) to track the relationship between early change and clinical outcome, comparing outcomes for patients in this study with benchmark data from a study that did not involve feedback about progress.

## Method

### Design

The design comprised an organizational intervention and implementation of a patient monitoring and feedback system framed within a practice-oriented research paradigm (Castonguay, Barkham, Lutz, & McAleavy, 2013). The outcome monitoring and feedback system comprised not only feedback on patient outcomes but also feedback on the alliance, social support, motivation, and stressful life events. In light of these additional components, we referred to this package as *enhanced* feedback. These components provided a focus for an evaluation of how therapists made use of this information. Therapists had access to outcome data throughout therapy but an enhanced feedback report was provided to therapists between the fourth and fifth sessions for each of their patients. A key design component of the study was the inclusion of two neighbouring but contrasting NHS services, one a smaller service and the other a larger and more complex service, in order to inform the extent to which complexity affected feasibility and acceptability.

Mixed methods were employed to investigate the feasibility, acceptability, and effectiveness of the patient monitoring and enhanced feedback system. There were seven main sources of data: (1) outcome measures completed by patients each session; (2) a therapist questionnaire measuring how they used the feedback; (3) a questionnaire measuring therapists’ experiences of the overall system; (4) therapists’ experiences of the system, expressed at review meetings; (5) a post-discharge questionnaire collecting patient experiences of the system; (6) feedback from patient focus groups; and (7) comparisons with benchmark data from a neighbouring service without a feedback system.

### Setting

Both services were based within the psychological therapies services of a large UK NHS Trust and provided therapy for patients with more severe, complex, and enduring mental health problems, including personality difficulties and interpersonal problems. The services were based in neighbouring metropolitan districts in West Yorkshire with both the smaller and larger services serving populations of about 200,000 people. Psychotherapeutic services are free to patients but require referral. The smaller service operated from a single base, with a single administrative support system, while the larger service operated from three substantial clinical bases: a health centre, a district general hospital, and a community centre. They will be referred to in this paper as the “small” and “large” services, respectively.

The new patient monitoring and enhanced feedback system was set up so that all therapists would participate, including trainees and temporary staff employed to deal with the waiting list in the larger service. A total of 31 therapists from the large service and 11 from the small service provided therapy for patients who consented to be included in the study. The total sample of 42 therapists comprised 26 permanent and qualified therapists, 8 trainee clinical psychologists, and 8 therapists either employed on a temporary basis to address the waiting list or who were based in another part of the service and provided short-term, sessional input. Of the 26 permanent and qualified therapists, 7 were cognitive behavioural therapists, 3 psychodynamic psychotherapists, and 16 clinical psychologists, whose main therapeutic orientations were psychodynamic, cognitive behaviour therapy (CBT), integrative psychotherapy, or cognitive analytic therapy. Some therapists worked part-time, so whole-time equivalents ranged from 0.2 to 1.0.

Similar types of therapy were provided in each service and mainly comprised psychodynamic psychotherapies and CBT. Group therapies were also provided within both services and focused on mood management and self-management skills, incorporating mindfulness and CBT approaches. In some cases, patients received more than one type of therapy (for example, individual followed by group therapy or vice versa). These are referred to as episodes of therapy. The large service underwent a major service re-organization during the course of the study, which introduced a triage system comprising a brief assessment/intervention of up to three sessions for a large number of patients who had been waiting a long time for treatment.

The benchmark data was collected as part of a previous and separate research study that included sessional outcome measurement, but without a feedback system (see Stulz et al., 2007). This service was in the same UK NHS Trust, in a neighbouring metropolitan district with a population of about 320,000.

### Measures

The study utilized a package of measures that tapped five areas: patient outcomes, process, clinical support tools, therapist feedback, and patients’ experiences. A hallmark of measure selection was that they should be brief and thereby enable the package of measures to have good bandwidth in terms of the breadth of data capture within routine service delivery.

#### Patient outcome (feedback sample)

CORE-10 (Barkham et al., 2013). The CORE-10 is a brief, pantheoretical questionnaire for routine use in practice settings and is a shortened version of the 34-item CORE-OM (Barkham et al., 2001; Evans et al., 2002). The CORE-10 taps 3 domains: (1) *Problems*: Depression (2 items), Anxiety (2 items), Physical (1 item), and Trauma (1 item); *Functioning*: General functioning (1 item), Social functioning (1 item), and Close relationships (1 item); and (3) *Risk*: To self (1 item). The CORE-10 does not tap the domain of subjective well-being that is included in the CORE-OM. As with the CORE-OM, the CORE-10 items are rated on a 5-point scale (0 = *not at all* to 4 = *most or all of the time*). The clinical score is computed as a mean of all the completed items, multiplied by 10 to give a possible range between 0 and 40. This is equivalent to a simple sum of the responses when all 10 items are completed. The CORE-10 has been shown to have good internal reliability (alpha) of 0.90 and a correlation with the CORE-OM of 0.94 in a clinical sample and 0.92 in a non-clinical sample and a 90% reliable change index of 6 (Barkham et al., 2013).

#### Patient outcome (benchmark comparison)

Two parallel 18-item short forms of the CORE-OM (CORE-SF-A & B, Barkham et al., 2001; Evans et al., 2002) representing the same three domains plus subjective well-being had been used for monitoring patient progress in the benchmark study, which preceded the development of the CORE-10. Each of these short forms comprises four items on subjective well-being, six on problems, six on functioning, and two detecting risk to self and others. The alpha coefficients for the two short forms are reported as .94 (Form A) and .94 (Form B) (Barkham et al., 2001). Items are rated on the same 5-point scale as the CORE-OM and CORE-10. The CORE-SF clinical score is computed as a mean of all the completed items, multiplied by 10 to give a possible range between 0 and 40, making it comparable to the CORE-10 scores.

#### Process measures

(1) The Assessment for Signal Cases (ASC; Lambert et al., 2007). The ASC comprises 40 items and taps patients’ functioning using a 5-point Likert scale with anchors of 1 (strongly disagree) to 5 (strongly agree). It was developed and used by Lambert and colleagues in feedback studies to help therapists identify problems and was linked to clinical support tools (Lambert et al., 2007). There are four subscales: therapeutic alliance (11 items), social support (11 items), motivation for therapy (9 items), and life events (9 items). A modified version of the ASC was used in the present study. The wording of eight items was modified, without changing the meaning, to take account of the UK context.^1^ For example, “I felt connected to a higher power” was replaced with “I have felt supported by religion/faith.” Responses to this questionnaire are not reported on here but it was an important component of the enhanced feedback after session 4. (2) Helpfulness Alliance and Stage Measure (HASQ). This is a 12-item brief measure with 1 item asking about the helpfulness of the session, 5 items assessing therapeutic alliance the short form Agnew Relationship Measure (ARM-5, Cahill et al., 2012), and a 6-item checklist assessing stage of change and based on Stiles’ assimilation model (Stiles, 2002). The HASQ also included a space for comments about the patient's current experience of therapy.

#### Feedback questionnaires

Three questionnaires relating to the feedback were used, two completed by the therapist and one by the patient.

The therapist questionnaires were as follows:

1. Feedback Response Questionnaire (FRQ) recorded how therapists responded to the enhanced feedback. This focused on how helpful the enhanced feedback report was, whether it reflected their view of the patient's progress, and how therapists viewed and responded to the report. They were asked to tick one or more of seven responses shown in Figure 2, for example, taking it to supervision or exploring feedback with the patient.
2. Feedback System Questionnaire (FSQ) included items on therapists’ experiences of the system and its impact on their practice. It included questions on interference with routine practice, helpfulness of various aspects of the feedback to therapy (e.g., the traffic light system, graphs, feedback reports), extent of access to graphs, helpfulness to risk assessment, assessment and formulation, developing a therapeutic alliance, and impact on aspects of the therapy provided (e.g., treatment goals, type of therapy, outcome, number of sessions). Items were rated on a 10-point scale. Each item offered the opportunity for therapists to make comments, which formed part of the data for thematic analysis. The questionnaire was completed by therapists prior to the review meetings and provided the structure for the meetings.
3. Patient Experience Questionnaire (PEQ) comprised seven statements tapping patients’ views and experiences of the patient monitoring and enhanced feedback system, including completing the process and outcome measures every session and their views regarding the impact on therapy. Items were rated on a 7-point scale from *strongly disagree* to *strongly agree*. The items are presented in Table IV.

### Procedure

The research proposal and related procedures were approved by the Bradford Research Ethics Committee (reference 10/H1302/80) as part of the National Research Ethics Service. All patients entering the larger and smaller services over an 18-month period were invited to take part in the study. Those who provided consent were asked to complete the CORE-10 (Barkham et al., 2013) before each session and the HASQ after each session. To support the enhanced assessment and feedback, the adapted form of the ASC (Lambert et al., 2007) was administered at session 4. All measures were completed in paper form and collected by the administrative and research team. The research team entered the ratings into a database.

#### Feedback procedure

**Figure 1.**
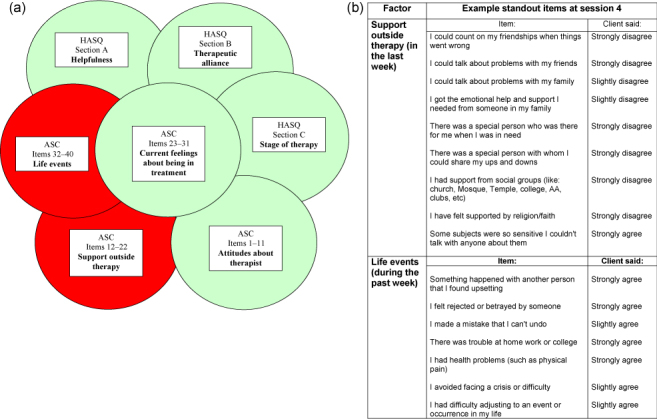
(a) Feedback report: Section reporting ASC and HASQ results. The red portions indicate the areas where your client may benefit from extra support. (b) Feedback report: Section reporting negatively rated ASC items (Red areas in Figure 1a).

Feedback on patient progress as recorded by the CORE-10 was provided in both services before session 5 in the form of a simple traffic light system indicating likely change: green indicating an improvement of 5 or more points by session 4, red indicating a deterioration of 5 or more points, and amber for those patients who had neither improved or deteriorated by 5 points or more. This 5-point change was informed by the reliable change index of 6 for the CORE-10 (Barkham et al., 2013) and was a pragmatic decision by the service to provide a simple, easily understood and over-inclusive criterion for judging whether a red feedback alert should be given. Where problems were highlighted on the ASC and HASQ, therapists were directed to sections of a therapist support manual, which included signposting to local support services. Figure 1(a) and 1(b) show an example of two sections from an enhanced feedback report for a patient whose CORE-10 scores had increased by 5 points at the fourth session. This patient had strong negative ratings on ASC items reflecting life events and social support. Figure 1(a) shows these ASC factors highlighted and the other factors on the ASC and HASQ not highlighted. Figure 1(b) shows the negatively rated items from the two ASC factors. In addition to the enhanced feedback after session 4, therapists had access to graphs on their patients’ progress (CORE-10 scores) throughout therapy via a shared network drive accessible from their desktop PCs.

### Feedback System

#### Therapists’ views

Therapists’ views about the system were collected at review meetings and from questionnaires. The review meetings were held at the mid-point of the 2-year study and towards the end of the study. Notes were taken at these meetings by the research team and key issues affecting the feasibility and acceptability of the system were discussed and agreed between all participants. Therapists also completed the FRQ after each enhanced feedback report had been received. Towards the end of the study, therapists completed the FSQ that provided information on their experience of the use and acceptability of the system.

#### Patients’ views

Patients who had received more than four sessions and whose therapist had received a feedback report were sent the PEQ by post after discharge and asked to return it by post. They were also asked to indicate their willingness to be involved in a focus group to provide more in-depth feedback on their experience of the system. Two focus groups were subsequently held, with seven patients attending one and five attending the other.

### Selection of Sample

**Figure 2.**
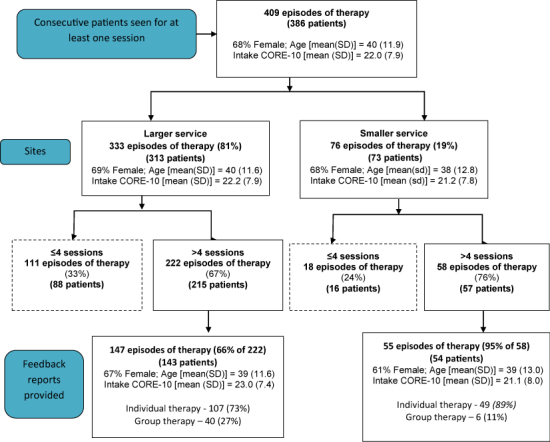
Flow diagram of patients within the two services.

Figure 2 presents a flow diagram charting the selection of the sample for which clinical outcomes were calculated. The total sample comprised 409 episodes of therapy, 333 delivered in the large service and 76 in the small service. An initial inclusion criterion for the study was that episodes of therapy included at least five therapy sessions, yielding 222 (larger service) and 58 (smaller service) episodes of therapy. A further criterion required episodes of therapy to include a CORE-10 completed at sessions 1 and 4, together with at least one more CORE-10 completed after session 4. These criteria ensured all clients could have received a feedback report indicating progress at session 4 with further outcome monitoring after session 4. This criterion reduced the sample to 202 episodes, 147 and 55 episodes for the large and small services, respectively. For this sample, 64% of patients were female, with a mean (SD) age of 39 years (12.0). In the benchmark sample, 69% of patients were female, with a mean (SD) age of 36 years (10.6).

### Analysis

#### Quantitative analysis

Patients were categorized as NOT (red traffic light) if CORE-10 scores had increased by 5 points or more between sessions 1 and 4; amber cases had neither improved nor deteriorated by more than 5 points; and green cases (categorized as “early responders”) had improved by 5 or more points. This is different from the definition of NOT used in the studies by Lambert et al. in which the term refers to patients who do not progress along expected recovery trajectories. Clinical outcomes and the relationship between progress at session 4 and final outcome were calculated for those patients whose therapists had received enhanced feedback and who attended at least five therapy sessions, representing a significant dose of therapy. The benchmark comparison group had also all attended at least five therapy sessions. Effect sizes were calculated by using study-specific means and SDs to yield separate effect sizes (ES) for the Feedback and Benchmark study. Comparisons between samples are reported using *t*-tests. When the Levene test for equality of variances indicated significant differences in variance, adjusted *t-*values and degrees of freedom are reported.

#### Qualitative analysis

The research team, comprising principal investigators and research assistants, identified initial themes (codes) from the notes taken at the first set of feedback meetings using thematic analysis (Braun & Clarke, 2006). These were then discussed with the therapists at the second round of meetings. Further thematic analysis incorporated written comments in the FSQ to ensure that all the overarching themes and subthemes were captured.

## Results

The rates of successfully providing feedback reports between sessions 4 and 5 are proxy measures of the success of the system in providing prompt feedback in each service. Reports were provided in 55 (95%) cases for the smaller service and 147 (66%) cases for the larger service (see lower portion of Figure 2).

### Intake Scores

Figure 2 also shows intake scores on the CORE-10 for the two services, with similar mean (SD) scores of 21.1 (8.0) for the smaller service and 23.0 (7.4) for the larger service. The overall mean intake CORE for the full sample of 202 (*M* = 22.5, SD = 7.6) is significantly larger than the mean intake CORE-SF for the benchmark sample (*N* = 136, *M* = 18.6, SD = 8.5; *t*(336) = 4.4, *p* < 0.001), but both the feedback and the benchmark sample means are well above the clinical cut-off for the CORE.

### Early Change Compared to Final Change

Table I shows the numbers and percentages of episodes of therapy that were categorized within the three traffic light categories at session 4 and whether they improved or deteriorated at the end of therapy. For patients in the Feedback sample, 60 (30%) showed early improvement of 5 or more points at session 4 (Green feedback), 116 (57%) showed no change at session 4 (Amber feedback), and 26 (13%) showed a deterioration of 5 or more points at session 4 (Red feedback). By the final session, 84 (42%) had improved at or beyond the reliable change index of 6 points for the CORE-10, 101 (50%) had shown no reliable improvement, and 17 (8%) had shown reliable deterioration.

**Table I.** Relationship between early and overall changes on the CORE-10 for study sample (with feedback) and benchmark (no feedback).

		Number (%) of patients meeting reliable change categories on the CORE-10 across therapy episodes		
Status at session 4	*N* (%)	Deteriorated ≥ 6 (Session 1 to last) *N* (%)	No change *N* (%)	Improved ≥ 6 (Session 1 to last) *N* (%)
*Current study (N = 202)*				
Not on track (Red)	26 (13)	9 (35)	12 (46)	5 (19)
No change (Amber)	116 (57)	8 (7)	73 (63)	35 (30)
Early responders (Green)	60 (30)	0 (0)	16 (27)	44 (73)
Totals		17 (8)	101 (50)	84 (42)
*Benchmark comparator (N = 136)*				
Not on track (Red)	12 (9)	3 (25)	7 (58)	2 (17)
No change (Amber)	86 (63)	9 (11)	55 (64)	22 (26)
Early responders (Green)	38 (28)	1 ( 3)	11 (29)	26 (68)
Totals		13 (10)	73 (54)	50 (37)

Of the 26 patients who had significantly deteriorated at session 4, 9 (35%) reliably deteriorated at final outcome while 5 (19%) reliably improved. The equivalent figures for the benchmark data were 3 (25%) and 2 (17%), respectively. Of the 60 early responders at session 4, none had significantly deteriorated at final outcome while 44 (73%) reliably improved. Of the 38 early responders in the benchmark data, 1 (3%) had significantly deteriorated at final outcome and 26 (68%) reliably improved. Overall, the proportions of patients in the different change categories at final outcome did not differ for the feedback study compared with the benchmark data (χ^2^(2, *N* = 338) = 0.81).

### Effect Sizes for the Feedback and Benchmark Samples

Pre–post effect sizes for the current study and the benchmark data are presented in Table II. Effect sizes (both positive and negative) are slightly, but non-significantly, larger for the current study (*M* = 0.56, SD = 1.00 for the feedback sample and *M* = 0.43, SD = 0.89 for the benchmark sample; *t*(336) = 1.25, *p* = 0.21). Table II also shows that both data sets show a negative effect size for the group who had deteriorated at session 4. In addition, the mean number of sessions was significantly higher for the Benchmark study (*M* = 20.1, SD = 17.2) than the Feedback study (*M* = 12.8, SD = 7.3; *t*(167.9) = 4.79, *p* < 0.001).

**Table II.** Effect sizes at end of therapy for each study by session 4 status.

Session 4 status	Feedback study (*n* = 202)	Benchmark study (*n* = 136)	ES(diff)
Green	1.28	1.03	0.25
Amber	0.36	0.24	0.12
Red	− 0.18	− 0.10	0.08
Overall	0.56	0.43	0.13

### Therapists’ Views on the Feedback System

Table III presents the results of the FSQ. This focused on therapists’ overall views of the service and was completed by 15 permanent, qualified therapists (4 from small service, 11 from large service) out of 24 who were working in the services at the time (7 from small service, 17 from larger service, 62.5% response rate). The main therapeutic orientations of these 15 therapists were CBT (*N* = 5), psychodynamic (*N* = 4), integrative (*N* = 5), and cognitive analytic therapy (*N* = 1). Seven (47%) therapists indicated they regularly accessed graphs and 8 (53%) indicated they did not access the graphs. A total of 13 (87%) therapists indicated that the feedback had not affected the number of sessions provided and 2 (13%) indicated that it led to fewer sessions being provided. Only 3 (20%) therapists agreed that feedback identified something they were not already aware of while 8 (53%) stated they specifically discussed the measures in supervision, and 5 (33%) therapists said they shared and discussed the feedback reports in supervision. In all, 9 (60%) therapists indicated they would like to continue with aspects of the feedback system as part of their routine therapy.

**Table III.** Therapists’ ratings on the FSQ (*n* = 15).

Items	Range	Mode (*N*)	Mean (SD)
Helpfulness (0 = *very unhelpful*; 10 = *very helpful*)			
How helpful have you found the feedback reports?	3–10	5 (5)	5.5 (2.0)
How helpful have you found the traffic light system?	2–10	7 (5)	6.1 (2.2)
How helpful were the feedback reports in relation to accessing risk?	0–8	5 (4)	4.7 (2.2)
How helpful was the feedback in relation to assessment and formulation?	2–8	7 (5)	5.4 (1.5)
Impact (0 = *not at all*; 10 = *very much*)			
How much did recruiting patients to the research interfere with normal therapeutic practice?	0–7	7 (4)	4.2 (2.5)
To what extent do you think the feedback reports reflect your patient's progress?	4–10	5 (4)	5.6 (1.6)
To what extent has the feedback affected treatment goals	0–8	0 (5)	2.1 (2.4)
To what extent has the feedback affected the type of therapy provided?	0–7	0 (9)	1.5 (2.4)
To what extent has the feedback affected the outcome of therapy?	0–5	0 (5)	1.5 (1.8)

**Figure 3.**
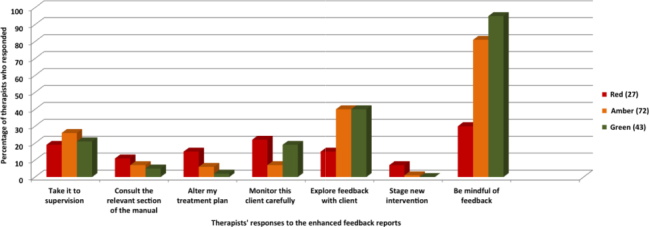
Therapists’ responses to feedback reports after session 4 for the three groups (*n* = 142).

In all, 142 FRQs were completed by therapists in relation to the enhanced feedback reports. A total of 115 (81%) reflected patients’ progress and 91 (64%) said the feedback report had been helpful to them and to their patient. Figure 3 shows the percentage of various actions resulting from receipt of an enhanced feedback report and indicates low levels of discussion of the feedback in supervision as well as in exploring it with patients. In addition, Figure 3 shows a very low incidence of changes to treatment and consulting the therapist support manual. For NOT patients, therapists tended to be less likely to explore the feedback with patients and less likely to find the feedback report helpful to the therapy they were offering.

The thematic analysis identified four themes from verbal and written feedback from the review meetings and the open questions of the FSQ. These themes were identified as affecting the feasibility, effectiveness, and acceptability of the system.

1. *The extent to which therapists integrated the measures and feedback into the therapy*. There was a range of experiences regarding the extent to which the outcomes monitoring and feedback system were found to be helpful to the therapeutic process. This view is supported by data presented in Table III, which shows the variability in ratings on the FSQ. Some therapists fully integrated the system into therapy and discussed the measures at each session. At least one therapist discussed each questionnaire item with patients at every session and found the measures and their use in therapy to be “invaluable.” Other therapists had concerns that the repeated measures were not helpful to the client or to the therapeutic relationship. There were also differences in the extent to which therapists were willing to use therapy time to complete the questionnaires with some strongly believing they should not be completed in the therapy room. At the review meetings, there was a broad agreement that therapists found that initial concerns about adverse effects on the therapy were reduced the more they became familiar with the system. However, a few therapists remained concerned about fundamental elements of the system such as whether the outcome measures could reflect the progress and the benefits patients gained from therapy.
2. *Availability of administrative support*. Therapists considered that the lack of administrative support adversely affected questionnaire completion, particularly in satellite clinic bases. This was also clear to the research team. Some therapists felt the resulting additional responsibility of ensuring complete data-sets detracted or impinged on their therapeutic time with the client. Lack of administrative support also adversely affected the timely administration of measures and ensuring therapist codes were attached to the questionnaires, which reduced the proportion of feedback reports successfully provided at the larger service.
3. *Information technology (IT).* IT problems adversely affected access to the graphs of the patients’ progress throughout therapy. The application containing the database and related graphs crashed on a number of occasions, which some therapists reported was a disincentive to routinely accessing the graphs.
4. *Complexity of the service and service changes*. These challenges were greater at the larger site and included the number of clinic bases, internal transfers of clients between episodes of therapy and therapists (e.g., when patients received both individual and group work), and service reorganizations. Immediately prior to the start of data collection, the large service also moved its main base with little warning. Attempts were made to track the changes in episodes of therapy but this was not always successful. Group work also presented challenges to the successful completion of measures at each session.

In addition to these themes, some therapists reported finding the system more helpful to therapy over time and there was a general view that the traffic light system was helpful and easily understood. Some therapists who were concerned that looking at the feedback reports would interfere with the therapeutic process came to see it as another useful channel of communication. Therapists expressed concerns about the concept of patients being NOT, when patients are going through a difficult stage in therapy such as exposure or trauma work. Therapists also reported that information on the alliance and on stresses in a person's life were particularly helpful, the latter helping set realistic treatment goals for people with very adverse life situations. A few therapists reported that some patients had found completion of the forms burdensome and were given “time off” from completing them. Therapists noted that some patients found it difficult to complete the post-session questionnaires after an intense session.

### Patients’ Views of the Feedback System

Table IV presents the mean ratings on the PEQ for 56 patients who had been discharged and their therapist had received an enhanced feedback report after session 4. The questionnaire was introduced a few months after the study began. It was sent to 179 patients, yielding a response rate of 31%. Table IV shows positive views of the system in terms of understanding why the forms were completed, ease of completion, and the time taken. Ratings were still positive, but less so on the questions relating to the impact on therapy.

**Table IV.** Patients’ ratings on the PEQ (*n* = 56).

Items	Range	Mode (*N*)	Mean (SD)
Experiences (1 = *strongly disagree*; 7 = *strongly agree*)			
I understand why I am being asked to fill in the forms	1–7	7 (36)	6.3 (1.3)
I find the forms easy to fill in	1–7	7 (23)	5.8 (1.4)
I am comfortable with the place in which I fill in the forms	1–7	7 (23)	5.9 (1.3)
The time it takes me to fill in the forms is acceptable	3–7	7 (29)	6.2 (1.0)
Filling in the forms has helped my therapy	1–7	6 (16)	5.2 (1.6)
The forms capture everything my therapist needs to know about my problems, progress, and therapy	1–7	7 (14)	5.1 (1.8)
My therapist uses information from my forms in the session	1–7	4 (14)	4.7 (2.0)

Patients’ views expressed at the two reference groups highlighted problems with the questionnaires, such as formatting and wording, and difficulties reflecting on the benefits of the therapy session immediately after an emotionally difficult session. However, there was general support for the idea of tracking progress using standardized questionnaires. Patients attending the reference groups all thought therapists should look at the responses to the questionnaire items but some questioned whether the therapists always did so.

## Discussion

The aims of this study were to investigate the feasibility and acceptability of an outcome monitoring and enhanced feedback system, what therapists did with the feedback, and the relationship between early change and outcome. We discuss each of these aims in turn.

In terms of our first aim concerning feasibility and acceptability, we highlight barriers and facilitators that can be categorized as operating at any one of three levels: patient level, therapist level, and service/organizational level. At the patient level, feedback from patients was, on the whole, positive. They saw the value of regular outcome monitoring, felt the measures were relevant, and did not find it too much of a burden, except for some concerns about completing questionnaires at the end of distressing therapy sessions. Feedback about improvements that could have been made to the layout of the forms supports the practice of involving service users in the research process (Brett, Staniszewska, & Mockford, 2010). It was also acknowledged that their involvement should have happened earlier in the research process.

At the therapist level, the main facilitator to engagement and a positive view of the system was the extent to which therapists integrated the measures and feedback into the therapy. This was, in turn, related to how compatible the therapists felt the system was to their own therapeutic approach. Youn, Kraus, and Castonguay (2012) argue that therapists may resist using outcomes monitoring and feedback systems because they believe it may interfere with the therapeutic alliance. A few therapists in our study did express this concern, although the study did not directly measure the impact on the alliance between the patient and therapist. We found no clear relationship between therapeutic orientation and integration with practice in that those therapists reporting integration practised CBT, psychodynamic therapy, and integrative therapy. Concerns about measures being collected too often and encroaching on the therapy time and space may be reduced with data inputting systems using hand-held computer devices and/or online, but for a few therapists, the objections to the system were more fundamental and attributable to a perceived incompatibility with their therapeutic approach. There is clearly evidence from this study of variability in the extent to which therapists took account of the feedback on their patients’ progress. Another possible factor influencing this variability is the extent to which they feel supported. A supportive culture is likely to facilitate reflective practice.

At the organizational level, the lack of availability of administrative support in some service bases, IT problems, and service complexity and reorganizations all adversely affected the acceptability, feasibility, and effectiveness of the system. The administrative support and IT problems could be mitigated with a more reliable system and such systems have been developed and reviewed (e.g., Barkham et al., 2010; Lutz, Bönke, & Köck, 2011). Systems involving inputting by patients on hand-held devices or online and linked to therapists’ computers provide efficient and prompt feedback to therapists and this is clearly the way forward. Ideally, these systems should also have the capability to capture the different episodes of therapy that may follow one another so that outcomes, and benchmarks of expected outcomes, relate to particular interventions. This is particularly important where both individual and group therapy are provided for patients and where stepped care service models are used. For example, in the UK, Improving Access to Psychological Therapies Services provide low-intensity interventions that may then be stepped up to a high-intensity therapy such as CBT, Counselling for Depression, or Interpersonal Therapy (Clark, 2011). Feedback systems should also be flexible enough to be used in group therapy. It is possible that patients in group therapy may be at particular risk of experiencing a deterioration that goes unnoticed by therapists.

The second aim was to look at what therapists do with feedback and this is one of the few studies to do that. Lutz et al. (2011) investigated responses of therapists to feedback in a German study and reported some action or reformulation as a result of feedback on about 70% of occasions. However, the percentages for most responses were relatively low with the highest—approximately 50%—relating to a discussion of the questionnaire answers with the patient. Our study also found low levels of the various responses to feedback. Of particular note was the finding that, for NOT patients, therapists tended to be less likely to explore the feedback with patients and to find the feedback report less helpful to therapy. The reasons for this finding are not clear. It may reflect a discomfort with negative feedback or a bias in therapists wishing to see their patients doing well and themselves as effective therapists (Walfish, McAlister, O'Donnell, & Lambert, 2012). In a similar vein, it is proposed that feedback works on the basis of a discrepancy between the feedback and the (ultimate) goal of patient improvement (e.g., De Jong, 2014). Accordingly, if that real or perceived discrepancy is not present, the utility and/or impact of feedback will be reduced. But this is only speculation. The clear implication of this finding is the need to understanding *how* therapists make sense of and respond to NOT feedback in particular and also to develop systems, methods, and a culture that encourages therapists to take account of feedback.

Lambert's studies suggest it is only the NOT patients whose outcomes improve with feedback. Hence, it is important to understand how therapists understand and respond to such feedback. The third aim was to understand how early change related to outcome and it is important to acknowledge that although progress at session 4 did predict outcome, this was not the case for all patients. Indeed, approximately 20% of patients who had reliably deteriorated at session 4 went on to record outcome scores meeting the criterion of reliable improvement. This is a small percentage and the numbers in this study were low, so this should not deflect from the clear message that early response is a predictor of outcome. However, it is important to acknowledge that a minority of patients may indeed feel worse before they improve. This phenomenon may be more prevalent with severe mental health problems as the early stages of therapy may involve elements such as becoming aware of and exploring distressing issues and exposure to traumatic memories. Acknowledgement of this phenomenon may help keep therapists engaged in the process and underlines the importance of acknowledging that any measure of change should be considered by therapists (and patients) in a broader context. On a similar theme, therapists found the information from the ASC scale helpful in that it provided some context with which to consider the patients’ progress, for example, for those with complex and adverse life situations.

The third aim also involves comparison of outcomes for this study with the benchmark study. Recovery and deterioration rates tended to be similar when based on the reliable change index. In both studies, deterioration rates were in the region of 8%–10% (Table I). These elevated rates are consistent with the Mohr (1995) review, which suggested higher deterioration rates with more severe and complex problems. It is important to point out that this deterioration rate was for patients receiving at least five therapy sessions. The deterioration rate was less for those receiving fewer sessions. There was a significant difference in the mean number of sessions provided in the two studies, with the patients in the current study receiving fewer sessions than in the benchmark study. Discussion within the service suggested the shorter length of therapy was due to changes towards a more managed service with more time-limited individual and group therapies being provided rather than the consequence of feedback.

It is important, however, to consider the impact of feedback on treatment length and the efficiency of the service. The service provided in the current study would seem to be more efficient in that similar outcomes were achieved with significantly fewer sessions. The method used to measure early deterioration was based on statistically reliable change, mirroring the criteria used for reliable improvement (Jacobson & Truax, 1991). However, we used a less stringent criterion than reliable change in order to identify patients at risk of a poor outcome. Indeed, it is probably too simplistic to view reliable deterioration as a mirror opposite of reliable improvement. There is a case for the criterion for deterioration to be less stringent—that is, more sensitive to indications of clinical deterioration rather than being viewed as no reliable change. In this context, we would support the approach taken by Lambert for monitoring system to flag up those patients who do not follow the expected course of improvement, particularly as these are the patients who appear to benefit most from feedback.

Several caveats need to be stated. First it is unclear the extent to which findings can be generalized to other service delivery systems. Although the services used were based in routine UK NHS settings, the diversity of services within the UK and internationally makes it unlikely that any single study could be representative of the wide range of services providing psychological therapy. Second, the study investigated a newly introduced feedback system and it is possible therapists would have been more positive and more responsive to the feedback with a more established system. However the study does highlight barriers and facilitators that will apply across other services and it is especially important to consider these issues in complex services providing therapy to patients with severe and complex problems.

In this context, Davidson et al. (2015) reviewed 10 feedback studies and two meta-analyses and questioned the generalizability of findings of existing feedback studies to other settings. They point out that the early studies by Lambert and colleagues were all carried out in a university counselling centre, predominantly with female students. Although further studies have found beneficial effects of feedback, the review reports *reduced* effect sizes in feedback studies with more severe mental health problems. Interestingly, such a tendency may dovetail with findings that therapist effects *increase* with greater patient severity (Saxon & Barkham, 2012). Indeed, there is the possibility that inherent variability across therapists—that is, therapist effects—moderates the activity of feedback per se. We certainly found evidence of considerable variability in therapists’ responses to utilizing feedback.

Reflecting on the combined weight of evidence, our study provides clear implications for the implementation of acceptable and effective monitoring and feedback systems. These include providing reliable information systems that can accommodate complexities such as working in different settings, individual and group therapy, and multiple episodes of therapy. They should also enable completion of patient-completed questionnaires outside the therapy space but with rapid or real-time access for therapists. Consideration should also be given to enabling patients to have direct access to progress graphs. These systems will be flexible enough to allow individual therapists to use the system in a way that suits their own therapeutic approach.

Overall, the findings add support to the body of evidence suggesting the positive impact of feedback systems and the acceptability of such systems to most patients and therapists. However, they also support the observations of Davidson et al. (2015) that more research on the implementation and effectiveness of feedback systems in more diverse patient settings and services is required. Similarly, we are mindful of the natural variability across therapists and would advocate that future research in the field of feedback takes account of this key phenomenon.

## Note

1. Permission was given to authors by Michael Lambert.
